# Prevalence of *pfmdr1*, *pfcrt*, *pfdhfr *and *pfdhps *mutations associated with drug resistance, in Luanda, Angola

**DOI:** 10.1186/1475-2875-7-236

**Published:** 2008-11-17

**Authors:** Paula Figueiredo, Carla Benchimol, Dinora Lopes, Luís Bernardino, Virgílio E do Rosário, Luís Varandas, Fátima Nogueira

**Affiliations:** 1 UEI Malária/Centro de Malária e Doenças Tropicais/IHMT/Universidade Nova de Lisboa, Rua da Junqueira, 96, 1349-008, Lisbon, Portugal; 2 Hospital Pediátrico Dr. David Bernardino, Av. Amílcar Cabral, Maianga, Luanda, Angola; 3 UEI Clínica das Doenças Tropicais/Centro de Malária e Doenças Tropicais/IHMT/Universidade Nova de Lisboa, Rua da Junqueira, 96, 1349-008, Lisbon, Portugal

## Abstract

**Background:**

Malaria is the infectious disease causing the highest morbidity and mortality in Angola and due to widespread chloroquine (CQ) resistance, the country has recently changed its first-line treatment recommendations for uncomplicated malaria, from CQ to artemisinin combination therapies (ACT) in adults, and sulphadoxine/pyrimethamine (S/P) in pregnant women. Loss of SP sensitivity is, however, progressing rapidly in Africa and, in this study, were investigated a number of molecular markers associated to CQ and S/P.

**Methods:**

Blood samples were collected from 245 children with uncomplicated malaria, admitted at the Pediatric Hospital Dr. David Bernardino (HPDB), Angola, and the occurrence of mutations in *Plasmodium falciparum *was investigated in the *pfmdr1 *(N86Y) and *pfcrt *(K76T) genes, associated with CQ resistance, as well as in *pfdhfr *(C59R) and *pfdhps *(K540E), conferring SP resistance.

**Results:**

The frequencies of *pfmdr1 *mutations in codon 86 were 28.6% N, 61.3% Y and 10.1% mixed infections (NY). The frequency of *pfcrt *mutations in codon 76 were 93.9% K, 5.7% T and 0.4% mixed infections (KT). For *pfdhfr *the results were in codon 59, 60.6% C, 20.6% R and 18.8% mixed infections (CR). Concerning *pfdhps*, 6.3% of the isolates were bearers of the mutation 540E and 5.4% mixed infections (K540E).

**Conclusion:**

The results of this epidemiologic study showed high presence of CQ resistance markers while for SP a much lower prevalence was detected for the markers under study.

## Background

Malaria is endemic throughout much of the Angolan territory, and is by far the highest cause of morbidity and mortality particularly among children under five years old and pregnant women. Malaria continues to be responsible for 50% of all outpatient attendance and around 25% of all hospital deaths . Recently and according to the Angola Malaria Indicator Survey, 2007,  these figures have been reduced due to control programmes, but about 35% of all cases and 70% of all deaths reported annually occur in children under five years of age.

The first cases of resistance to chloroquine (CQ) were described in the eighties [1-4]. As a consequence of increasing rates of clinical resistance to CQ, the protocol at the Pediatric Hospital David Bernardino (HPDB) was changed, in 2006, two combined therapies artesunate with lumefantrine (Coartem^®^), and amodiaquine (AQ) with sulphadoxine-pyrimethamine (SP – Fansidar^®^) as first-line treatments of uncomplicated malaria.

The *pfmdr1 *gene (multidrug resistance) has been described as associated with CQ resistance [5-7], with a polymorphism resulting from the substitution of an asparagine for a tyrosine in amino acid 86 (N86Y) in *Plasmodium falciparum *[8-14]. CQ resistance is also associated with a mutation in the transporter gene *pfcrt *where the amino acid substitution at *pfcrt *codon 76 (K to T) has been shown to have a determinant association with the resistance phenotype [15-19].

*Plasmodium falciparum *resistance to sulphadoxine and pyrimethamine (SP) is conferred by mutations of the dihydropteroate synthase (*pfdhps*) and dihydrofolate reductase (*pfdhfr*) genes, respectively [20-23]. *pfdhfr *108N mutation seems to be enough to confer resistance to pyrimethamine [24,25]. The presence of a mutation at positions 51 (N51I) or 59 (C59R), together with S108N confer a considerable increase in the resistance level to pyrimethamine when compared with mutation S108N by itself [26-28].

Mutations in codons 437 (A437G) and 540 (K540E) of *P. falciparum pfdhps *are associated with resistance to sulphadoxine. The quintuple mutant (triple *pfdhfr*: 51I, 59R, 108N and double *pfdhps*: 437G, 540E) is considered as the molecular marker of SP treatment failure [29-34]. Studies of genetic transfection of *P. falciparum*, confirmed that the amino acid substitution at *pfdhfr *codon 108 (S→N), increases approximately ten times the resistance to pyrimethamine [35]. Epidemiology studies also demonstrated that the presence of mutations 59R in *pfdhfr *and 540E in *pfdhps *are significantly associated to resistance as well as to the presence of the other above mentioned mutations [30,36,37].

In this work were investigated the frequencies of mutations associated with chloroquine resistance (*pfmdr*1 N86Y and *pfcrt *K76T) and also screened for the two mutations associated to the resistance of *P. falciparum *to SP as used in populations from high malaria transmission areas.

## Materials and methods

### Sample collection

Blood samples included in this study were collected from children between 1 and 16 years, at the HPDB Hospital in Luanda, Angola, and individually spotted on Whatman n.°4 filter paper, after microscopic confirmation of *P. falciparum *infection. Parent's informed consent was obtained before inclusion of the blood samples in the study which was reviewed and approved by the Ethical Committees from the Ministry of Health of Angola and of the HPDB. Original study was associated to quinine treatment and published elsewhere [38].

### Genetic characterization of the parasites

Parasite DNA was extracted from dried blood spots, using Chelex as described elsewhere [39]. *P. falciparum *mutations associated with resistance to CQ and SP were typed by PCR-RFLP as described elsewhere [39,40], primers sequences, amplification cycles and restrictions enzymes are described in table 1 and 2, respectively. In this study, the following codons and polymorphisms were analysed: *pfmdr1 *86, *pfcrt *76; *pfdhfr *59 and *pfdhps *540. Amplicons and fragments were separated on 2% or 3% agarose gels stained with ethidium bromide and visualized under UV.

**Table 1:** PCR Programme for the genes *pfmdr*1 86, *pfcrt *76, *pfdhps *540, *pfdhfr *59, primer and product size

**Gene**	**Primers**	**PCR Programme**			**Product size (bp)**
*Pfmdr*1 86	754N 754R	Initial step	92°C	3 min	321
		Denaturation (35×)	92°C	1 min	
		Annealing (35×)	51°C	30 s	
		Extension (35×)	72°C	1 min	
		Final step	72°C	3 min	
*Pfcrt *76 1° nested	76o1F N1R	Initial step	94°C	3 min	528
		Denaturation (40×)	94°C	45 s	
		Annealing (40×)	55°C	45 s	
		Extension (40×)	72°C	45 s	
		Final step	72°C	3 min	
*Pfcrt *76 2° nested	76 N2F 76 N2R	Initial step	94°C	3 min	271
		Denaturation (35×)	94°C	45 s	
		Annealing (35×)	53°C	45 s	
		Extension (35×)	72°C	45 s	
		Final step	72°C	3 min	
*Pfdhps *540	540 F 540 R	Initial step	94°C	3 min	439
		Denaturation (40×)	94°C	1 min	
		Annealing (40×)	45°C	1 min	
		Extension (40×)	72°C	1 min	
		Final step	72°C	3 min	
*Pfdhfr *59	59 F 59 R	Initial step	94°C	3 min	326
		Denaturation (35×)	94°C	1 min	
		Annealing (35×)	55°C	1 min	
		Extension (35×)	72°C	1 min	
		Final step	72°C	3 min	

**Table 2:** Fragments length, clones and enzymes used for digestion of the codons 86 *Pfmdr *1, 76 *Pfcrt*, 540 *Pfdhps *and 59 *Pfdhfr*.

Gene	Enzyme	clone	Codon	Fragments length
*Pfmdr1 *86	*Apo*I	3D7	AAT (Asn)	249 + 72 bp
		Dd2	TAT (Tyr)	321 bp
*Pfcrt *76	*Apo*I	3D7	AAA (Lys)	137 + 124 + 10 bp
		K1	ACA (Thr)	261 + 10 bp
*Pfdhps *540	*BseG*I	N3	GAA (Glu)	354 + 85 bp
		T9/94	AAA (Lys)	439 bp
*Pfdhfr *59	*Pdm*I	K1	CGT (Arg)	354 + 85 bp
		HB3	TGT (Cys)	439 bp

### Statistical analysis

Associations between the different mutations were tested by Fisher's exact test.

## Results

### Patients and parasites

**Figure 1:**
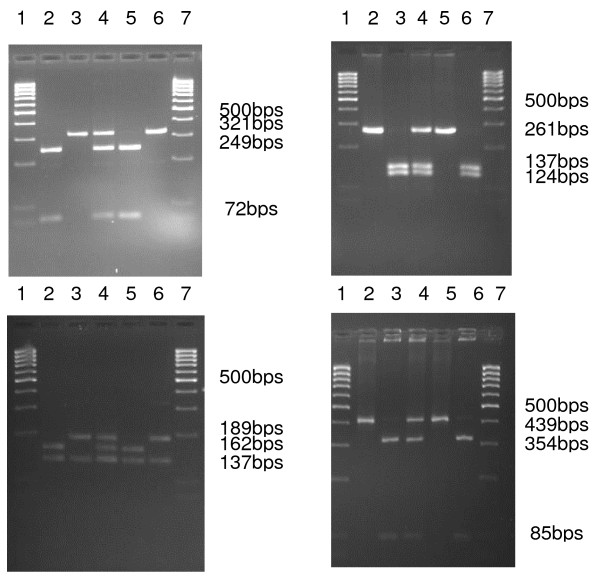
**Agarose gels showing *pfmdr*1 86, *pfcrt *76, *pfdhfr *59 and *pfdhps *540 PCR-RFLP products of control and field-collected samples of *P. falciparum*.*** pfmdr1*: 2-N86; 3-Y86; 4-N/Y86; 5-3D7 (N86); 6-Dd2 (Y86). *pfcrt*: 2-k76; 3-T76; 4-K/T76; 5-K1 (K76); 6-3D7 (T76). *pfdhfr*: 2-C59; 3-R59; 4-R/C59; 5-N3 (C59); 6-T9/94 (R59). *pfdhps*: 2-E540; 3-K540; 4-K/E540; 5-HB3 (E540); 6-K1 (K540). In all gels lanes 1 and 7 shows the molecular weight marker.

From the 245 isolates of *P. falciparum *parasites used in this study, 199 samples were successfully typed by PCR-RFLP for *pfmdr*1, for *pfcrt *245, for *pfdhps *221 and for *pfdhfr *224. Mutant alleles were detected in four loci: *pfcrt *76T, *pfmdr1 *86Y, *pfdhps *540E and *pfdhfr *59R (Figure 1).

#### *pfmdr1 *and *pfcrt*

As shown in Table 3, the frequency of the pure mutant allele *pfcrt *T76 was 93.9%. Only one isolate carried a mixed population KT for this allele (0.41%). The frequency of the mutant *pfmdr1 *Y86 allele was 61.3% and mixed *pfmdr1 *N and Y allele was detected in 10.1% of the isolates.

**Table 3:** Prevalence of mutations conferring resistance to chloroquine and sulphadoxine/pyrimethamine in *Plasmodium falciparum *isolates from Angola

Gene	**Mutation**	n	**Mutation (%)**	Mixed* (%)
*Pfcrt*	T76	245	93.9 (230/245)	0.4 (1/245)
*pfmdr1*	Y86	199	61.3 (122/199)	10.1 (20/199)
*pfdhfr*	R59	224	20.6 (46/224)	18.8 (42/224)
*pfdhps*	E540	221	6.3 (14/221)	5.4 (12/221)

*refers to mixed populations of *P. falciparum *parasites.

To study the associations between the loci *pfcrt *K76T and *pfmdr1 *N86Y, 176 single infection isolates were analysed. Among the 165 isolates with the *pfcrt *T76 mutation, 115 (70%) also carried the mutant *pfmdr1 *Y86 allele. Whether or not the mixed infections were excluded from the analysis, the association between the two mutant alleles was significant (*P *= 0.029, mixed infections excluded).

#### *pfdhps *and *pfdhfr*

One locus of *pfdhps *(K540E) and one of *pfdhfr *(C59R) were investigated (Table 3). For the *pfdhps *88.3% of the isolates carried the mutant allele E540 and 5.4% carried mixed (K and E) alleles. For *pfdhfr *60.6% were wild type (C59) and 18.8% were mixed populations. To study the associations between the *pfdhps *E540 and *pfdhfr *R59 loci, only 61 single infection isolates were analysed, and there was no association between *pfdhps *E540 and *pfdhfr *R59 (*P *= 0.159).

## Discussion

*In vivo *resistance to CQ and SP have reached high levels in some regions of Angola, where resistance to these drugs is high (25%) [41]. This study reveals a high frequency of drug resistance molecular markers for, CQ (*pfcrt *T76; 93.9% and *pfmdr1 *Y86; 61.3%) and SP (9% of bearers with quintuple mutant).

In this study, associations within and between the mutations that confer resistance against CQ and those considered predictive of SP treatment failure were evaluated. Obtained data revealed an association (*P *= 0.029) between *pfcrt *T76 (chromosome 7) and *pfmdr1 *Y86 (chromosome 5) as reported by other authors [42-45].

Presence of mutations R59 and E540 in *pfdhfr *and *pfdhps*, respectively, is considered a marker of confirmed resistance to SP and our data indicates that 9% of the children attending the HPDB, were bearers of *P. falciparum *parasites with the SP quintuple mutant associated usually to treatment failure. These results are in line with others from western Africa, where low prevalence of the quintuple mutant has been observed [46,47]. Although the predictive value of these markers for SP treatment failure has not been established in this study, these results emphasize the need for close monitoring of mutation prevalence with treatment outcome, since these antimalarials are now used as first line treatment of uncomplicated malaria in children attending the HPDB as well as prophylactic treatment in pregnant women.

Inter- and intragenic association of *pfdhfr *and *pfdhps *mutant codons was indirectly proven in other studies where SP resistance was found to be associated with double up to quintuple mutations in both genes [48,49]. Here, only 9% of the samples, with concurrence of the *pfdhfr *and *pfdhps *variants, R59 and E450 simultaneously were observed, which are considered to be predictive of the quintuple mutant (*pfdhfr *I51, R59, N108, *pfdhps *G437, E540) associated to SP treatment failure [37,50-53]. All other samples had presence of one or the other mutation suggesting that fixation of the quintuple mutant was still an on going process.

Association between mutations on *pfcrt*, *pfmdr1*, *pfdhfr *and *pfdhps*, have been reported from West Africa [54,55] but reports on linkage between response to treatment with CQ and SP, in the same patients, is rare. Even though no statistic association was found, 43 double mutants for *pfdhfr/pfcrt*, 21 for *pfdhfr/pfmdr1*, 12 *pfdhps/pfcrt *and 6 *pfdhps/pfmdr1 *were detected. Regarding the association between mutations conferring resistance to SP and CQ; almost all of the isolates carrying the mutant genotype for *pfdhfr *(43 out of 46) or *pfdhps *(12 out of 14) carried at least one of the CQ associated mutations in the genes *pfcrt *or *pfmdr1*. This reflects the possible association and accumulation of at least three or four out of 7 mutations (*pfcrt *T76, *pfmdr*1 Y86, *pfdhfr: *N108, I51, R59 and *pfdhps: *G437, E540) scattered on four different chromosomes and involved in resistance to three different antimalarials, chloroquine, sulfadoxine and pyrimethamine.

As a result of this study it is not possible to comment whether the presence of *pfcrt *T76 favors the presence of *pfdhfr and pfdhps *mutations or *vice versa *because this requires longitudinal and long lasting studies and observations. Despite these difficulties in drawing firm conclusions, several previous findings support the hypothesis of linkage disequilibrium between mutations associated with SP resistance and CQ resistance. In a murine malaria model, CQ resistance could be induced in pyrimethamine-resistant parasites, but not in sensitive ones [56].

In field isolates, several observations indicate that it is more likely to find mutations in *pfdhfr *and *pfdhps *in isolates exhibiting already *pfcrt *T76 mutation than in isolates comprising *pfcrt *wild type parasites [57-59]. The reason for this apparent association between resistance to CQ and SP is obscure since both drugs have distinct modes of action and resistance to these is determined by mutations on different chromosomes [60-62]. The accelerated acquisition of resistance to multiple drugs (ARMD) possibly reflecting a rapid mutator phenotype [63] could be one explanation.

Multiple CQ and SP resistance mutations are thought to have higher fitness cost of the parasite asexual reproduction (reviewed in [64,65]). In line with the previous considerations, the reproductive capacity of mutant strains is believed to be jeopardized [64-66]. The results did not reflect these since gametocyte prevalence in infections with mutant-type parasites, was not higher than in infections with wild-type isolates.

## Conclusion

This was the first molecular study carried out in this geographical area including a considerable number of samples (245) and focused in the mutations of *pfmdr*1, *pfcrt*, *pfdhps *and *pfdhfr *genes, strongly associated to CQ and SP resistance. The results of the epidemiological study on prevalence of genotypes associated with drug resistance, carried out at a HPDB in Luanda, showed high presence of CQ resistance markers, while for SP a much lower prevalence was detected. This work can be important to evaluate the implementation of new therapeutics strategies based on combinations that includes SP, like the protocol that are now implemented in HPDB (artesunate combined with SP) as first-line drug for uncomplicated malaria treatment.

## Competing interests

The authors declare that they have no competing interests

## Authors' contributions

PF carried out the molecular analyses and drafted this manuscript. FN participated in the design of the study performed the statistical analysis and helped to draft the manuscript. CB carried out the selection of children and sample collection. DL helped in molecular analysis and the draft of the manuscript. LB coordinated sample collection in PHDB as hospital director. VEdR helped in coordination and design of the study. LV coordinated the project and designing of the study. All authors read and approved the final manuscript.
